# Predictive factors of nivolumab plus ipilimumab treatment efficacy in metastatic renal cell carcinoma patients

**DOI:** 10.1093/jjco/hyae046

**Published:** 2024-04-22

**Authors:** Kojiro Ohba, Hiromi Nakanishi, Ken Kawada, Yuichiro Nakamura, Kensuke Mitsunari, Tomohiro Matsuo, Yasushi Mochizuki, Ryoichi Imamura

**Affiliations:** Department of Urology and Renal Transplantation, Nagasaki University Hospital, Nagasaki, Japan; Department of Urology and Renal Transplantation, Nagasaki University Hospital, Nagasaki, Japan; Department of Urology and Renal Transplantation, Nagasaki University Hospital, Nagasaki, Japan; Department of Urology and Renal Transplantation, Nagasaki University Hospital, Nagasaki, Japan; Department of Urology and Renal Transplantation, Nagasaki University Hospital, Nagasaki, Japan; Department of Urology and Renal Transplantation, Nagasaki University Hospital, Nagasaki, Japan; Department of Urology and Renal Transplantation, Nagasaki University Hospital, Nagasaki, Japan; Department of Urology and Renal Transplantation, Nagasaki University Hospital, Nagasaki, Japan

**Keywords:** renal cell carcinoma, nivolumab, ipilimumab, predictive factor, paraneoplastic symptom

## Abstract

**Objective:**

Nivolumab plus ipilimumab is a recommended first-line therapy regimen for metastatic renal cell carcinoma. However, it is not clear which patient characteristics are associated with its effectiveness.

**Methods:**

We retrospectively examined 67 metastatic renal cell carcinoma patients treated with nivolumab plus ipilimumab as a first-line therapy in multiple institutions from September 2018 to August 2022. We analyzed the relationships between survival outcomes and patient-related variables, including paraneoplastic symptoms. We also analyzed the relationships between changes in symptoms and parameters and outcomes.

**Results:**

Of the 67 patients, 32 patients had paraneoplastic symptoms. The median progression-free survival was 14.9 months and median overall survival was 43.3 months. The objective response rate was 49.25% (33 patients), including two patients with complete response. Patients with cytoreductive nephrectomy, bone metastasis, high C-reactive protein levels and paraneoplastic symptoms were significantly correlated with short progression-free survival in the univariate analysis. Multivariate analysis of these factors showed that the presence of paraneoplastic symptoms at treatment initiation remained an independent predictor of progression-free survival. Of the 32 patients with paraneoplastic symptoms at treatment initiation, 12 patients had symptomatic improvement and 20 did not. The 1-year progression-free survival rates were significantly longer in improved patients compared with those with no improvement.

**Conclusions:**

Patients without cytoreductive nephrectomy and with bone metastasis, liver metastasis, high C-reactive protein levels and paraneoplastic symptoms were significantly correlated with shorter progression-free survival. The presence of paraneoplastic symptoms was an independent predictor of progression-free survival. Improvement in paraneoplastic symptoms may reflect the treatment efficacy of nivolumab plus ipilimumab.

## Teaser

Having paraneoplastic symptoms was an independent predictor of progression-free survival for the patients with metastatic renal cell carcinoma, and symptom improvement may reflect the treatment efficacy of nivolumab plus ipilimumab.

## Introduction

For metastatic renal cell carcinoma (mRCC), the currently recommended first-line treatment regimen is a combination of immune checkpoint inhibitors (ICIs) or an ICI in combination with another therapeutic. Recently, several randomized phase 3 trials were conducted to assess potential first-line treatment regimens for mRCC and have been recommended (1–5). These regimens were superior when compared with tyrosine kinase inhibitor (TKI) monotherapy, but no comparisons were made between these regimens. Combination regimens include two types of ICI together or an ICI combined with a TKI. Currently, there is no clear standard for selecting a double ICI combination or an ICI and TKI combination regimen.

In the TKI era, the prognosis of mRCC patients has been stratified by the International Metastatic Renal Cell Carcinoma Database Consortium (IMDC) risk classification (6). In ICI combination regimens, using a programmed death (PD)-1 inhibitor (nivolumab) with a cytotoxic T-lymphocyte-associated protein 4 (CTLA-4) inhibitor (ipilimumab) is the only reported one that has used double ICIs (1).^1^ This regimen has included 4 years minimum of follow-up data and continues to demonstrate a durable efficacy benefit (7). However, this study showed that 19.3% of patients treated with nivolumab plus ipilimumab had no therapeutic effect, compelling us to take caution before selecting this regimen for mRCC patients. Nivolumab plus ipilimumab has been a recommended regimen for mRCC patients in IMDC intermediate and poor risk groups (1).^1^ However, some ICI plus TKI regimens have also been suggested for these patients (2–5). The variety of treatment options may overwhelm physicians who are trying to choose the most appropriate treatment approach. Therefore, it is necessary to clarify which subsets of patients would potentially benefit from such regimens.

Although several subgroup analyses have been reported, the specific characteristics that would make nivolumab plus ipilimumab effective for mRCC patients remain unclear (1,7,8). Although some studies have analyzed real-world data, no information has been obtained that is useful for patient selection (9–11). Furthermore, no biomarkers have been identified that could be used to determine potentially effective regimens. It is also unknown which patients are not suitable for nivolumab plus ipilimumab treatment. It is especially difficult to select a treatment plan for patients who appear to have a poor prognosis, such as those with paraneoplastic symptoms.

In this study, we aimed to investigate the specific patient profile associated with effective treatment using nivolumab plus ipilimumab, and to assess the relationship between changes in paraneoplastic symptoms and outcomes.

## Patients and methods

This study was approved by the Human Ethics Review Committee of Nagasaki University Hospital (Nagasaki, Japan; No. 18101527) and was performed in accordance with the ethical principles of the Declaration of Helsinki. We retrospectively examined 67 patients with mRCC. These patients were treated for recurrent, metastatic or unresectable disease with nivolumab plus ipilimumab as the first-line therapy in multiple institutions. The inclusion criteria were as follows: (i) no prior systemic therapy, (ii) available clinical data and (iii) began treatment from September 2018 to August 2022. Patients enrolled in this study had a minimum observation period of 6 months, with a median time of 15.2 months.

In the induction, nivolumab was administered at a dose of 240 mg and ipilimumab was administered at a dose of 1 mg/kg intravenously on the same day. After completing four administrations in the induction phase, nivolumab alone was administered every 2 weeks at a dose of 240 mg or every 4 weeks at a dose of 480 mg.

Treatment response was assessed by computed tomography, which was regularly performed at 4- to 12-week intervals depending on patient conditions. Efficacy was evaluated according to the Response Evaluation Criteria in Solid Tumors (RESIST) version 1.1 (12). Objective response rate (ORR) was defined as the sum of the complete response (CR) and partial response (PR) rates. Progression-free survival (PFS) and overall survival (OS) were defined as the time from initiation of nivolumab plus ipilimumab treatment to progression of disease (evaluated by RESIST) and death, respectively.

We then analyzed the relationships between PFS/OS and patient-related variables, such as patient characteristics, clinicopathological features, the IMDC risk classification, paraneoplastic symptoms and inflammatory parameters. We also analyzed the relationships between changes in symptoms and parameters and outcomes.

### Statistical analysis

Continuous data are presented as the median and range, and categorical data are presented as patient count and percentage. To compare the patient-related variables, the Mann–Whitney U and chi-square tests were used for continuous and categorical data, respectively. Kaplan–Meier analysis was used to assess PFS and OS, with comparisons made using the log-rank test. Cox proportional hazard models were used to identify parameters that are correlated with PFS or OS, and the risk was expressed as hazard ratios (HRs) and 95% confidence intervals (CIs). Parameters with *P* < 0.05 in the univariate analysis were included in the multivariate analysis. Two-tailed *P* < 0.05 was accepted as statistical significance. Statistical analyses were performed using SPSS Statistics for Windows, version 19.0 (IBM Corp, Armonk, NY, USA).

## Results

### Patient characteristics

Overall, 67 patients were enrolled in this study, with a median age of 70 years (range: 21–89). Among these, 32 patients had paraneoplastic symptoms and 16 patients had a Karnofsky Performance Status (KPS) of <80% (Table 1). Details of the paraneoplastic symptoms are shown in Table 2. The histological type was unknown in 11 patients, while 10 patients had sarcomatoid features.

**Table 1:** Metastatic renal cell carcinoma (mRCC) patient characteristics

	*N* = 67
Age, years (median and range)	70 (21–89)
Male	49 (73.1%)
Prior nephrectomy	35 (52.2%)
Histology	
Clear	46 (68.7%)
Non-clear	10 (14.9%)
Unknown	11 (16.4%)
With sarcomatoid features	10 (14.9%)
Bone metastasis	19 (28.4%)
Liver metastasis	5 (7.5%)
Paraneoplastic symptoms	32 (47.8%)
From diagnosis to systemic therapy <1 year	58 (86.6%)
KPS < 80	16 (23.9%)
Hb < LLN	29 (43.3%)
Ca > ULN	13 (19.4%)
Neutrophils > ULN	18 (26.9%)
Platelets > ULN	20 (29.9%)
CRP > 1.0	33 (49.3%)
NLR > 3.0	32 (47.8%)
IMDC	
Intermediate	38 (56.7%)
Poor	29 (43.3%)

KPS, Karnofsky performance score; LLN, lower limit of normal; ULN, upper limit of normal; CRP, C-reactive protein; NLR, neutrophil to lymphocyte ratio; IMDC, International Metastatic Renal Cell Carcinoma Database Consortium.

**Table 2:** Types of paraneoplastic symptoms

	*N* = 67
Pain	18 (26.9%)
Anorexia	5 (7.5%)
Hemorrhage	4 (6.0%)
Paralysis	4 (6.0%)
Fatigue	4 (6.0%)
Fever	3 (4.5%)
Dyspnea	3 (4.5%)
Weight loss	2 (3.0%)

### Treatment outcomes

**Figure 1:**
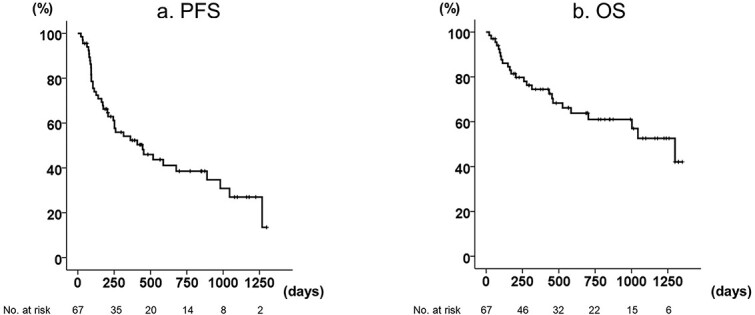
Survival outcomes of 67 patients treated with nivolumab plus ipilimumab. Kaplan–Meier plots of (a) progression-free survival (PFS) and (b) overall survival (OS).

Of the 67 patients, 50 were able to receive four doses of ipilimumab. Patients discontinued this treatment because of adverse events in 21 cases and progression of disease in 24 cases. After nivolumab plus ipilimumab treatment, 20 patients received TKIs, including 11 with cabozantinib, 8 with axitinib, 3 with sunitinib, 5 with pazopanib and 1 with sorafenib. Some of these 20 patients received more than one TKI. Twenty-five patients died from disease progression. The 1- and 2-year PFS rates were 52.3 and 38.5%, respectively, and the median PFS was 14.9 months (95% CI: 5.8–23.8, Fig. 1a). The 2- and 3-year OS rates were 61.0 and 52.6%, respectively, and the median OS was 43.3 months (95% CI: 28.5–58.1, Fig. 1b). Analysis of pretreatment factors and PFS revealed that patients without cytoreductive nephrectomy (CN), and with bone metastasis, high C-reactive protein (CRP) levels and paraneoplastic symptoms at the initiation of ipilimumab and nivolumab treatment were significantly correlated with short PFS in the univariate analysis (Table 3). After multivariate analysis of these putative prognostic factors, paraneoplastic symptoms at the initiation of ipilimumab and nivolumab treatment (HR: 2.358, 95% CI: 1.098–5.050, *P* = 0.028) remained as an independent predictor of PFS. For the OS univariate analysis, advanced age, the absence of CN and the presence of sarcomatoid features, bone metastasis, paraneoplastic symptoms, high CRP and high neutrophil to lymphocyte ratio (NLR) at the initiation of ipilimumab and nivolumab treatment were significantly correlated with short OS (Table 4). As shown in Table 5, the ORR was 49.25% (33 patients) including two patients (2.99%) with CR, but no pretreatment factors were significantly correlated with ORR.

**Table 3:** Univariate and multivariate Cox regression analyses for progression-free survival (PFS) in the overall population and according to the baseline patient characteristics

	Univariate			Multivariate		
	HR	95% CI	*P* value	HR	95% CI	*P* value
Age > 75 years	1.095	0.533–2.252	0.804			
CN	0.429	0.226–0.814	0.01	0.725	0.349–1.508	0.399
Non-clear cell	1.752	0.781–3.931	0.174			
Sarcomatoid features	1.039	0.424–2.546	0.933			
Bone metastasis	2.121	1.073–4.193	0.031	1.866	0.917–3.788	0.086
Paraneoplastic symptoms	3.125	1.623–6.024	0.001	2.358	1.098–5.050	0.028
CRP > 1.0	1.978	1.049–3.731	0.035	1.506	0.759–2.985	0.241
NLR > 3.0	1.807	0.966–3.378	0.064			
IMDC poor risk	1.297	0.951–1.770	0.101			

CI, confidence interval; CN, cytoreductive nephrectomy.

**Table 4:** Univariate Cox regression analyses for overall survival (OS) in the overall population and according to the baseline patient characteristics

	Univariate		
	HR	95% CI	*P* value
Age > 75 years	3.515	1.576–7.841	0.002
CN	0.364	0.156–0.846	0.019
Non-clear cell	1.981	0.710–5.528	0.192
Sarcomatoid features	2.888	1.092–7.636	0.033
Bone metastasis	2.777	1.241–6.212	0.013
Paraneoplastic symptoms	2.818	1.261–6.296	0.012
CRP > 1.0	3.692	1.536–8.873	0.004
NLR > 3.0	2.315	1.032–5.192	0.042
IMDC poor risk	1.340	0.905–1.988	0.144

**Table 5:** Best confirmed objective response rate (ORR) in the overall population

	*N* = 67	(%)
CR	2	2.99
PR	31	46.27
SD	18	26.87
PD	16	23.88

CR, complete response; PR, partial response; SD, stable disease; PD, progression disease.

### Changes in paraneoplastic symptoms and outcomes

Following these results, we investigated whether the treatment efficacy could be predicted with a change in paraneoplastic symptoms. Of the 32 patients with paraneoplastic symptoms at the initiation of ipilimumab and nivolumab treatment, 12 patients had symptomatic improvement and 20 did not.

**Figure 2:**
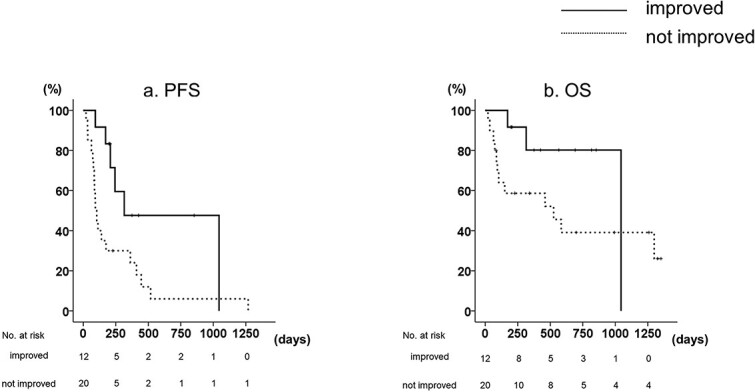
Survival outcomes of 32 patients who had paraneoplastic symptoms treated with nivolumab plus ipilimumab. Kaplan–Meier plots of (a) PFS and (b) OS.

The 1-year PFS rates were significantly longer in patients with symptomatic improvement compared with those without improvement (47.6 vs. 24.0%, *P* = 0.020, Fig. 2a). A similar trend was observed with the 2-year OS rates, but there was no statistically significant difference between the groups (80.2 vs. 39.1%, *P* = 0.133, Fig. 2b). There was no significant association between NLR or CRP reduction and symptom improvement, nor was PFS significantly different with NLR or CRP reduction.

## Discussion

In many mRCC cases, either double ICI or ICI plus TKI is selected as first-line treatment. However, there are currently no clear criteria for deciding which treatment to choose. To our knowledge, no biomarkers have been identified that can predict the efficacy of ICI combination therapies. Therefore, the treatment strategy for mRCC is not unified, and treatment selection can be an overwhelming process. For these reasons, it is necessary to identify predictors of treatment efficacy among patient characteristics before and during treatment.

In this study, we analyzed pretreatment patient characteristics to predict the efficacy of the nivolumab plus ipilimumab regimen. The results suggest that PFS was significantly shorter in patients with paraneoplastic symptoms at the initiation of treatment than in those without symptoms. Paraneoplastic symptoms with RCC are known to include pain, fever, fatigue, weight loss and blood test abnormalities, such as neutrophilia, eosinophilia, thrombocytosis, anemia or hypercalcemia (13–15).

Until the TKI era, patients with paraneoplastic symptoms were already known to have a poor prognosis (16–18). The current study shows that prognosis with combined immunotherapy is also poor in symptomatic patients. Additionally, patients without CN, bone metastasis, and high CRP levels at the initiation of ipilimumab and nivolumab treatment had shorter PFS. A systematic review showed that CN had a role in improving prognosis in patients with mRCC (19). However, another interpretation is that CN could only be performed in selected patients who were expected to have good prognosis. For other factors, there were some reports that patients with bone or liver metastasis before treatment also had poor outcomes (20,21). But in the randomized, phase 3 CheckMate 214 trial, patients treated with nivolumab plus ipilimumab had better prognoses than those treated with sunitinib, which was the standard regimen in the TKI era for bone and/or liver metastases (1). Our study indicated no significant difference in PFS by patient age or IMDC risk factors. Generally, it has been suggested that immune function declines with age (22). However, double ICI treatment efficacy has been observed in elderly as well as younger patients. In the TKI era, patients with more IMDC risk factors had a worse prognosis (6), whereas the ORRs were similar regardless of the number of IMDC risk factors in the CheckMate 214 trial (23). Collectively, this suggests that treatment selection in the ICI era should be judged based on factors other than IMDC risk factors and age.

Interestingly, among symptomatic patients, the prognosis was better in those who showed paraneoplastic symptomatic improvement after treatment with nivolumab plus ipilimumab compared with those who did not show symptomatic improvement. Overall, 37.5% of symptomatic patients showed symptom improvement, and about half of these improved patients had a sustained response to treatment. To the best of our knowledge, there have been no reports that analyzed paraneoplastic symptomatic improvement and prognosis in patients treated with nivolumab plus ipilimumab. Monitoring improvements in paraneoplastic symptoms provides an opportunity to determine whether treatment has been effective before assessing the patient via imaging. Depending on the patient’s status, it may also be possible to avoid missing an opportunity to alter their treatment by monitoring changes in paraneoplastic symptoms, which could be useful when done during daily clinical practice. In recent years, there has been some debate about whether to avoid using nivolumab plus ipilimumab in patients with paraneoplastic symptoms (24,25). The findings of this study are relevant to this.

Reduced NLR and CRP were not significant prognostic factors, potentially because these changes are not specific to mRCC. NLR and CRP may change not only with symptoms, but also with other factors, such as adverse events or infections. It seems difficult to predict the efficacy of nivolumab plus ipilimumab by NLR and CRP factors. However, some reports have shown that high baseline values of the NLR and CRP were associated with poor outcomes (26,27), while other reports suggested that the improvement of CRP levels possibly affected better outcomes (28,29). There has been no report on the relationship between NLR changes and prediction of treatment outcome. Further prospective studies are needed to clarify these findings.

This study has some limitations. First, it is a retrospective study with a small sample size and short follow-up periods. Therefore, a durable response, which is said to be a feature of nivolumab plus ipilimumab, could not be determined here (30). Second, immortal time bias occurred regarding opportunities for paraneoplastic symptom improvement caused by differences in prognosis. To resolve this, landmark analysis or time-dependent analysis is necessary. However, the number of patients with paraneoplastic symptoms was not sufficient for these analyses to be conducted and the follow-up period was too short. Additionally, the intentions of the attending physician and restrictions on the use of other regimens at each facility could possibly lead to a selection bias. To clarify the prognostic factors, including changes in symptoms, a large prospective clinical study should be conducted.

## Conclusions

In conclusion, we investigated efficacy prediction for mRCC patients treated with nivolumab plus ipilimumab. This study showed that patients without CN and with bone metastasis, liver metastasis, high CRP levels and paraneoplastic symptoms were significantly correlated with PFS. The presence of paraneoplastic symptoms was an independent predictor of PFS. Furthermore, improvement in paraneoplastic symptoms may reflect the treatment efficacy of nivolumab plus ipilimumab.
